# Reporting two novel *Kluyvera* species, *Kluyvera huaxiensis* and *Kluyvera chengduensis*, isolated from human sputa

**DOI:** 10.3389/fmicb.2025.1683098

**Published:** 2026-01-05

**Authors:** Lina Liu, Yu Feng, Hongxia Wen, Zhiyong Zong

**Affiliations:** 1 Center of Infectious Diseases, West China Hospital, Sichuan University, Chengdu, China; 2 Laboratory of Pathogen Research, West China Hospital, Sichuan University, Chengdu, China; 3 Division of Infectious Diseases, State Key Laboratory of Biotherapy, Chengdu, China; 4 State Key Laboratory of Respiratory Health and Multimorbidity, Chengdu, China

**Keywords:** *Kluyvera huaxiensis* sp. nov., *Kluyvera chengduensis* sp. nov., 16S rRNA gene sequence, genome sequence, taxonomy

## Abstract

**Introduction:**

Two Gram-stain negative, facultatively anaerobic, rod-shaped, motile, and non-spore forming bacterial strains, 142053^T^ and 142359^T^, were isolated from sputum samples obtained from different patients.

**Methods:**

Growth of strain 142053^T^ and 142359^T^ occurred at 8–42 °C (optimum 28–37 °C), pH 5.0–9.0 (optimum 6.0–8.0) and NaCl concentrations of 0–5% (w/v) (optimum 0–4%). Comparison of 16S rRNA gene sequences showed that strain 142053^T^ and 142359^T^ exhibited high similarity to *Kluyvera intermedia* NBRC 102594^T^ (99.48%) and *Kluyvera georgiana* ATCC 51603^T^ (98.68%), respectively.

**Results:**

Phylogenomic tree of species from family *Enterobacteriaceae* revealed that strain 142053^T^ and 142359^T^ formed distinct branches within the *Kluyvera* clade. The digital DNA–DNA hybridization between 142053^T^, 142359^T^, and all type strains of *Kluyvera* species were ≤39.5% and ≤54.7%, respectively, and the corresponding average nucleotide identity were ≤90.56% and ≤93.85%, both below the species delineation threshold. The two strains differ phenotypically from other *Kluyvera* species by their lipase and arginine dihydrolase utilization and from each other by ᴅ-arabitol utilization. The predominant fatty acids identified in both strains were C_16:0_, summed feature 3 (C_16:1_*ω7c*/C_16:1_*ω6c*), summed feature 8 (C_18:1_*ω7c*) and C_17:0_ cyclo.

**Discussion:**

Based on the phenotypic, chemotaxonomic, phylogenetic and genomic evidence, strains 142053^T^ and 142359^T^ represent two novel species of the genus *Kluyvera*, for which the names *Kluyvera huaxiensis* sp. nov. and *Kluyvera chengduensis* sp. nov. are proposed, with type strains 142053^T^ (= GDMCC 1.4845^T^ = JCM 37413^T^) and 142359^T^ (= GDMCC 1.5094^T^ = JCM 37737^T^), respectively.

## Introduction

*Kluyvera* is a genus of Gram-negative, rod-shaped bacteria in the family *Enterobacteriaceae* (Farmer et al., 1981). Currently, the genus *Kluyvera* contains five validly published species-*Kluyvera ascorbata*, *Kluyvera cryocrescens*, *Kluyvera georgiana*, *Kluyvera intermedia*, and *Kluyvera sichuanensi*s and five unnamed genomospecies (1, 2, 3, 4/6, and 5) (Farmer et al., 1981; Müller et al., 1996; Pavan et al., 2005; Liu et al., 2020; Rodriguez and Gutkind, 2024). *Kluyvera* typically inhabits sewage, hospital sinks, and human sputum, urine, or the gastrointestinal tract (Farmer et al., 1981; Sarria et al., 2001; Li et al., 2019; Alsuheili et al., 2024; Endo et al., 2024). Currently, there is an increasing number of reports on human infections caused by this bacterium, including oral infections, bloodstream infections, enteritis, biliary tract infections, abdominal abscesses, and urinary tract infections. Clinically, these infections pose challenges such as therapeutic difficulty, prolonged treatment duration, and adverse outcomes, including patient death (Mutoh et al., 2019; Zou et al., 2019; Akiki et al., 2023; Inoue et al., 2023; Ulloa-Clavijo et al., 2023). The most frequently reported *Kluyvera* species associated with human infections are *K. ascorbata* and *K. cryocrescens* (Wong, 1987; Lin et al., 2002; Yoshino et al., 2016; Zou et al., 2019), although clinical cases involving *K. intermedia* and *K. georgiana* have also been reported in recent years (Inoue et al., 2023; Endo et al., 2024; Xu et al., 2025). *Kluyvera* possesses a large and significantly flexible gene pool (Yu et al., 2023). Notably, the extended-spectrum *β*-lactamase gene *bla*_CTX-M_ and the fosfomycin resistance gene *fosA* originated from this genus (Humeniuk et al., 2002; Rodríguez et al., 2004; Ito et al., 2018; Rodriguez et al., 2018). Alarmingly, several plasmid-borne carbapenemase genes, including *bla*_KPC_ (Tiwari et al., 2022), *bla*_OXA-48_ (Hernández-García et al., 2018), and *bla*_NDM_ (Loiodice et al., 2023), and colistin resistance genes *mcr* (Strepis et al., 2021) have also been identified in *Kluyvera* species. Taken together, *Kluyvera* represents an emerging clinical and epidemiological concern due to its dual role as an opportunistic pathogen and as a reservoir of antimicrobial resistance genes. In this study, we conducted a taxonomic analysis on two clinical strains, 142053^T^ and 142359^T^, which were found to represent two novel species within the genus *Kluyvera*.

## Materials and methods

### Strains and the study

Strain 142053^T^ and 142359^T^ were isolated from the sputum samples of two different inpatients at West China Hospital in Chengdu (30.6800°N, 104.0811°E), China, in April and August 2024, respectively. The two sputum samples were collected as part of routine care for managing suspected pulmonary infection in the two patients. The sputum samples were vortexed with 0.9% normal saline at a 1:1 ratio for 10 s. The mucoid portion was then picked up with a sterile cotton swab and inoculated onto 5% sheep blood agar (Huankai; Guangzhou, China) plates using the quadrant streak method. The plates were incubated at 37 °C for 48 h, and the colonies of the two isolates were round, smooth, convex, and white. The two isolates were stored long-term at −80 °C in nutrient broth (Huankai) containing 20% glycerol. Preliminary species identification was conducted using matrix-assisted laser desorption ionization time of flight mass spectrum (MALDI-TOF; Bruker; Billerica, MA) with the MBT Compass Library 2023 according to the manufacturer’s instructions. The study has been approved by the Ethical Committee of West China Hospital with waiver of informed consent as this study was to characterize bacterial strains.

### Analysis of the 16S rRNA gene sequence

Genomic DNA of strain 142053^T^ and 142359^T^ was extracted using a bacterial genomic DNA extraction kit (Tiangen; Beijing, China). The 16S rRNA gene was amplified using universal primers 27F and 1492R (Moreno et al., 2002), and sequenced bidirectionally using Sanger sequencing (Sangon Biotech; Shanghai, China) for preliminary taxonomic identification. The assembled 16S rRNA gene sequences were compared with the NCBI 16S rRNA database using BLAST, applying a 98% identity threshold to identify closely related genera. Along with those retrieved from type strains of closely related genera, 16S rRNA gene sequences of 142053^T^ and 142359^T^ were aligned using Clustal Omega v1.2.4 (Madeira et al., 2019).

### Whole genome sequencing and analysis

Genomic DNA was extracted using the same method as described above. Whole genome sequencing of strain 142053^T^ and 142359^T^ was performed by NovaSeq 6,000 system (Illumina; San Diego, CA, USA). Reads were *de novo* assembled into contigs using SPAdes v4.0.0 (Bankevich et al., 2012) in isolate modes. The draft genomes of strain 142053^T^ and 142359^T^ were compared with those of type strains of *Kluyvera* species to assess genomic relatedness using average nucleotide identity (ANI) and *in silico* DNA–DNA hybridization (*is*DDH). ANI and *is*DDH values were calculated using FastANI v1.34 (Jain et al., 2018), OrthoANI (Yoon et al., 2017) and Genome-to-Genome Distance Calculator (formula 2) (Meier-Kolthoff et al., 2013b) with the default settings, respectively. POCP was calculated as described previously (Qin et al., 2014). Sequences were submitted to the online TYGS platform^1^ to assess their identification at the genus or species level (Meier-Kolthoff and Göker, 2019).

Genome sequences of type strains of all *Enterobacteriaceae* species were retrieved from GenBank. These sequences were annotated using Prokka v1.14.5 (Seemann, 2014). Orthologues were identified via PIRATE v1.0.5 (Bayliss et al., 2019) to represent the core genome. A maximum-likelihood (ML) phylogenetic tree was inferred from core-genome using IQ-tree v2.1.2 (Minh et al., 2020) with the GTR + GAMMA model and 1,000 bootstrap replicates. Additionally, neighbor-joining (NJ) and maximum parsimony (MP) trees were constructed with 1,000 bootstrap replicates (Kumar et al., 2018) to assess the robustness of the phylogeny. All genomic data analyzed in this study are available in Supplementary 1. Antimicrobial resistance determinants were predicted using ABRicate v1.0.0^2^ against ResFinder databases. Plasmid replicon types were determined using the PlasmidFinder tool available from the Center for Genomic Epidemiology.^3^

### Physiology and chemotaxonomy

Cell motility was examined by observing the bacterial growth and diffusion on the deep semi-solid nutrient medium of 0.3% (w/v) agar (Hopebio; Qingdao, China). Anaerobic growth was examined by streaking the bacterial cultures on brain heart infusion agar plates and placed in the GasPakTM EZ anaerobic bag (BD; Franklin Lakes, NJ, USA) at 37 °C for 3 days. After incubation in nutrient broth at 37 °C for 3 days, the flagella of strain 142053^T^ and 142359^T^ were observed using an H-7650 transmission electron microscope (Hitachi; Tokyo, Japan). Bacterial growth was examined in 5 mL aliquots of nutrient broth. These aliquots were dispensed into tubes with an inner diameter of 16 mm and incubated at temperatures of 4, 8, 18, 28, 32, 37, 42, 45, 48, and 50 °C for 7 days. Meanwhile, salt and pH tolerances were measured using nutrient broth at 37 °C for 7 days at different NaCl concentrations (0, 0.5, 1, 2, 3, 4, 5, 7.5, 10, and 15%, w/v) and at a pH unit of 4.0–12.0 (in increments of 1.0 unit), respectively (Hu et al., 2017). Catalase activity was tested by examining the production of bubbles after addition of 3% (v/v) hydrogen peroxide solution and oxidase activity was measured by using 1% tetramethyl-p-phenylenediamine dihydrochloride solution.

DNase activity was detected using DNase agar (Solarbio; Beijing, China). After incubation at 30 °C for 3 days, 1 M HCl was added for the detection. Malonate, phenylalanine deaminase, and KCN experiments were carried out using biochemical identification tubes (Huankai). Commercially available API 20E, API 50CH, and API ZYM kits (bioMérieux; Marcy l’Etoile, France) were employed to determine biochemical characteristics and enzyme activities. The assays were conducted in accordance with the manufacturer’s instructions, with *Escherichia coli* strain ATCC 25922 and *Pseudomonas aeruginosa* strain ATCC 27853 serving as controls. All experiments were performed in triplicate.

### Fatty acid analysis

Cellular fatty acid analysis was conducted by the Guangdong Institute of Microbiology (Guangdong, China). In brief, bacteria were inoculated onto nutrient agar plates and incubated at 37 °C for 24 h. Fatty acid methyl esters were extracted and then analyzed via gas chromatography following the guidelines of the Sherlock Microbial Identification System (MIDI Inc.; Newark, DE, USA), as detailed in previous reports (Sasser, 1990). The peaks were automatically integrated, and the proportions of fatty acids were calculated using the MIDI identification database RTSBA6 (version 6.00; MIDI Inc.).

### Antimicrobial susceptibility testing

Minimum inhibitory concentrations (MICs) of amikacin, ampicillin, ampicillin-sulbactam, aztreonam, ceftriaxone, ceftazidime, cefepime, cefotaxime, cefuroxime, chloramphenicol, ciprofloxacin, colistin, imipenem, meropenem, piperacillin-tazobactam, sulfamethoxazole-trimethoprim, and tigecycline against these two isolates was determined using the broth microdilution method in accordance with the guidelines of the Clinical and Laboratory Standards Institute (CLSI) (CLSI, 2025). Cation Adjusted Mueller-Hinton Broth (Oxoid, Basingstoke, UK) was used, and antimicrobial powders (purity ≥98%) were purchased from Meilunbio (Dalian, China) or Macklin (Shanghai, China). The breakpoints for colistin and tigecycline were interpreted based on the criteria of the European Committee on Antimicrobial Susceptibility Testing (EUCAST);^4^ otherwise, the breakpoints defined by the CLSI were applied.

## Results

### Two novel *Kluyvera* species were identified

The 16S rRNA gene sequence of strain 142053^T^ showed highest similarity to that of *K. intermedia* NBRC 102594^T^ (99.48%) and *Citrobacter freundii* ATCC 8090^T^ (99.22%), while that of strain 142359^T^ was most similar to *K. georgiana* ATCC 51603^T^ (98.68%) and *Klebsiella electrica* DSM 102253^T^ (98.11%). A maximum-likelihood tree was constructed based on 16S rRNA gene sequences, incorporating the sequences of the genera *Citrobacter, Klebsiella*, and *Pseudocitrobacter*, which are closely related to *Kluyvera* (Liu et al., 2020). Both strains clustered within the *Kluyvera* clade. However, they each form relatively long branches distinct from other *Kluyvera* species, suggesting potential taxonomic novelty (Supplementary Figure S1). Given the limited resolution of 16S rRNA gene-based analyses for differentiating species within the *Enterobacteriaceae* (Church et al., 2020), whole-genome sequencing was performed to confirm their phylogenomic placement and assess species-level distinctiveness.

Sequencing of strain 142053^T^ yielded 11,580,316 paired-end reads, representing 1.74 Gb of data, with a Q30 score of 93.5% and an average quality score of 26.8. These reads were then assembled into 80 contigs (N*50* = 183,280 bp). The draft genome of strain 142053^T^ is 5,003,793 bp in size, with a G + C content of 53.49 mol%. Strain 142359^T^ produced 8,187,706 reads, representing 1.23 Gb of data, with a Q30 score of 95% and an average quality score of 29.2, which were assembled into 52 contigs (N*50* = 341,410 bp). The draft genome of strain 142359^T^ is 4,907,633 bp in size, with a G + C content of 54.48 mol%. The POCP values for strains 142053^T^ and 142359^T^ against the reference strains of all *Kluyvera* species ranged from 75.82 to 87.03% and from 76.49 to 86.15%, respectively, supporting their assignment to the genus *Kluyvera*. The ANI values between strain 142053^T^ and the type strains of all other *Kluyvera* species ranged from 85.80 to 90.56%, while strain 142359^T^ showed ANI values between 85.45 and 93.85% relative to these type strains. All values were below the ≥95–96% ANI threshold recommended for species delineation (Meier-Kolthoff et al., 2013b) (Table 1). The OrthoANI values were also below the 95% (Supplementary Figure S2). Correspondingly, the *is*DDH values between strain 142053^T^ and other *Kluyvera* type strains ranged from 28.8 to 39.5%, and those for strain 142359^T^ ranged from 27.8 to 54.7%, well below the ≥ 70.0% threshold for species delineation (Richter and Rossello-Mora, 2009; Meier-Kolthoff et al., 2013a). Notably, strain 142053^T^ exhibited high genomic relatedness to the unnamed *Kluyvera* genomospecies 5, with *is*DDH and ANI values of 87.4 and 98.39%, respectively (Table 1). These results indicate that *K.* genomospecies 5 and *K. huaxiensis* represent the same species. Therefore, *K.* genomospecies 5 should be formally designated as *K. huaxiensis.*

**Table 1:** Average nucleotide identity (ANI), *in silico* DNA–DNA hybridization (*is*DDH) and POCP values between strain 142053^T^, 142359^T^ and the reference strains of other *Kluyvera* species.

Species	References strain	Accession no.	ANI (%)		*is*DDH (%)		POCP (%)	
			142053^T^	142359^T^	142053^T^	142359^T^	142053^T^	142359^T^
*K. ascorbata*	ATCC 33433^T^	JMPL01	85.98	93.85	28.9	54.7	80.51	86.15
*K. sichuanensis*	KCTC 82166^T^	JABBJF01	86.30	91.31	29.7	42.8	78.30	80.47
*K. georgiana*	ATCC 51603^T^	LXEU01	85.80	87.09	28.8	30.9	79.02	78.38
*K. cryocrescens*	NBRC 102467^T^	BCTM01	90.56	85.63	39.5	28.4	86.31	82.82
*K. intermedia*	ATCC 33110^T^	BCYS01	86.66	85.45	30.4	27.8	75.82	76.49
Genomospecies 1	L2	LGHZ01	89.69	85.58	37.4	28.3	87.03	84.03
Genomospecies 2	KA2	PYHO01	85.54	86.61	28.2	30.1	78.54	79.77
Genomospecies 3	PO2S7	CP050321	85.75	87.01	28.5	30.5	81.44	83.69
Genomospecies 4/6	D51-sc-1712206	ERR2221162	86.08	85.55	29.7	27.9	76.79	79.17
Genomospecies 5	169	SRR13099670	98.39	86.23	87.4	29.1	85.09	77.93
*K. huaxiensis*	142053^T^	JBEFLV01	–	86.04	–	29.0	–	80.57
*K. chengduensis*	142359^T^	JBIQOI01	86.04	–	29.0	–	80.57	–

**Figure 1:**
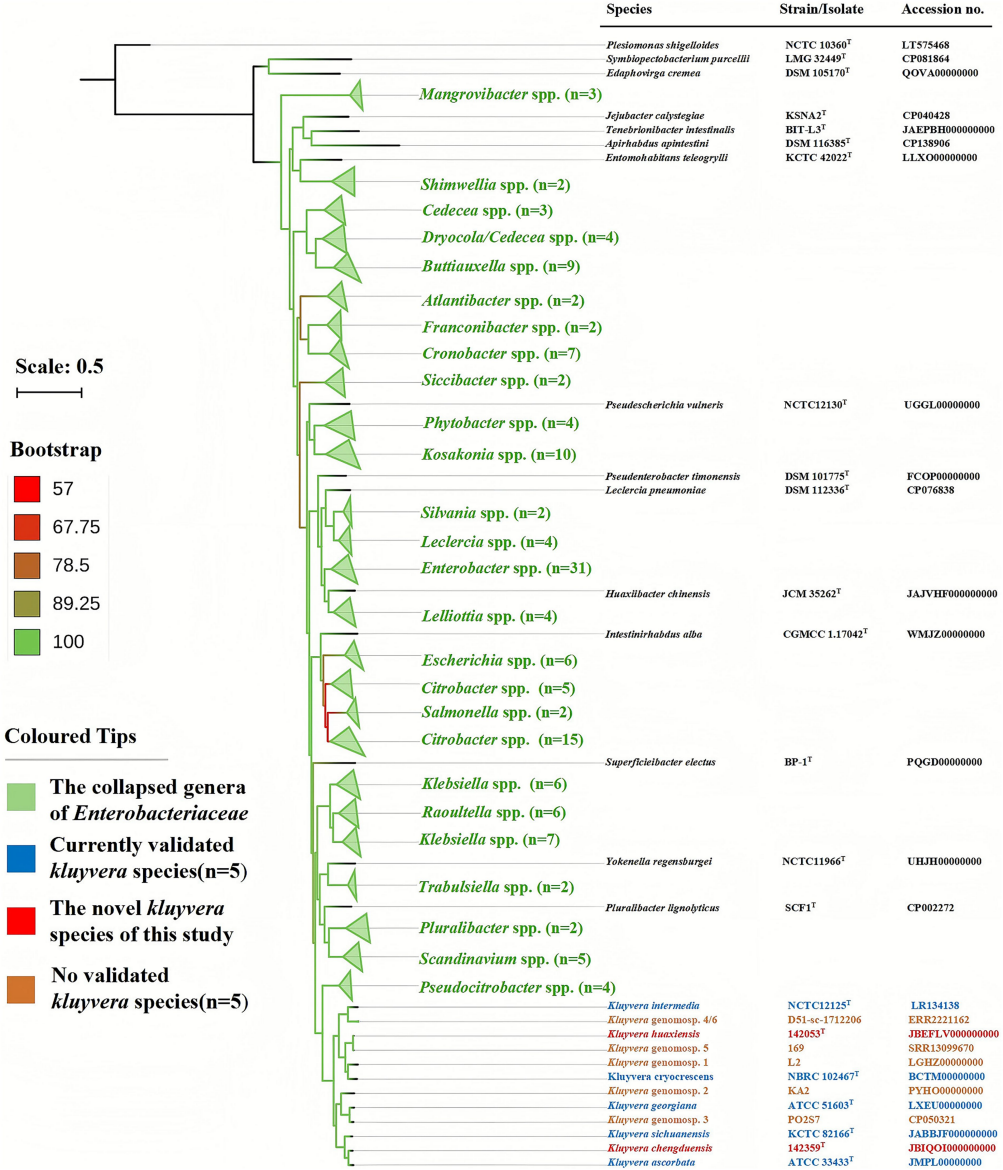
Phylogenomic tree of strain 142053^T^, 142359^T^, and other genera in the family *Enterobacteriaceae*. The core genome tree was constructed via the maximum-likelihood method based on 1,000 resamplings. Bar, 0.5 substitutions per nucleotide position.

**Figure 2:**
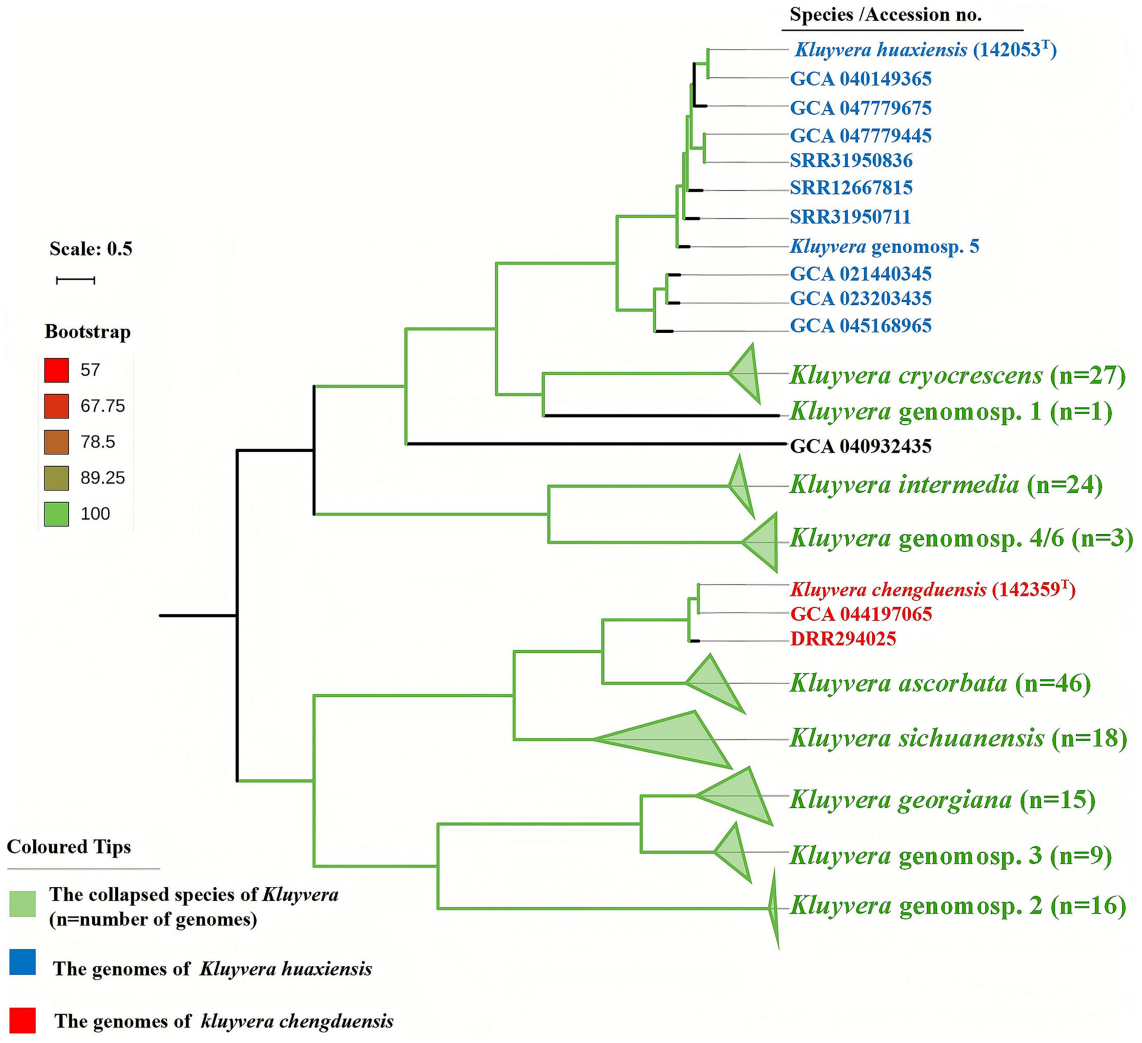
Phylogenomic tree of strain 142053^T^, 142359^T^, and other species in the genera *Kluyvera*. The core genome tree was constructed via the maximum-likelihood method based on 1,000 resamplings. Bar, 0.5 substitutions per nucleotide position.

A maximum-likelihood phylogenetic tree constructed using 265 core genes from the family *Enterobacteriaceae* (Figure 1) placed both strain 142053^T^ and 142359^T^ within a clade comprising members of genus *Kluyvera*. For independent validation, NJ, MP and TYGS-based phylogenomic trees were reconstructed (Supplementary Figures S3–S5), and the results were consistent with the ML phylogeny. This *Kluyvera* clade was well separated from other related genera, including the most closely related genus *Pseudocitrobacter*, further supporting that strain 142053^T^ and 142359^T^ belong to the genus *Kluyvera*. The ML phylogeny inferred from 1,668 core genes within the genus *Kluyvera* positioned strains 142053^T^ and 142359^T^ in two distinct clades, supporting their classification as novel species, as both were clearly separated from all currently recognized *Kluyvera* species (Figure 2). Taken together, these genomic and phylogenomic analyses provide robust evidence that strains 142053^T^ and 142359^T^ represent two novel species within the genus *Kluyvera*.

The physiological and biochemical characteristics of strain 142053^T^ and 142359^T^ are listed in Table 2. Briefly, both strains are positive for indole production, nitrate reduction, citrate utilization, and the activities of lysine decarboxylase, arginine dihydrolase, ornithine decarboxylase, *β*-galactosidase (ONPG), and catalase. These strains can assimilate aesculin, amygdalin, ᴅ-cellobiose, ᴅ-glucose, ᴅ-galactose, ᴅ-lactose, ᴅ-maltose, ᴅ-melibiose, ᴅ-sorbitol, ᴅ-trehalose, ᴅ-xylose, ʟ-arabinose, ʟ-rhamnose, mannitol, malonate, glycerol, methyl *α*-ᴅ-glucopyranoside, and salicin. Conversely, they are negative for acetoin production (Voges-Proskauer), DNase activity, H_2_S production, oxidase activity, phenylalanine deaminase activity, and the activities of gelatinase and urease. Moreover, neither strain can utilize adonitol, dulcitol, erythritol, or inositol. In comparison with other species in genus *Kluyvera*, strains 142053^T^ and 142359^T^ display unique physiological characteristics. Both show weakly positive lipase activity and are positive for arginine dihydrolase. Additionally, the two strains can be distinguished from each another by ᴅ-arabitol utilization, with 142359^T^ but not 142053^T^ can metabolize ᴅ-arabitol. These distinct traits serve as markers for differentiating strains 142053^T^ and 142359^T^ from other *Kluyvera* species. The cellular fatty acid profiles of two novel strains were analyzed and compared with that of *K. sichuanensis* GDMCC 1.1872^T^ (Supplementary Table S1). The fatty acid composition of other *Kluyvera* species has not yet been reported. The major cellular fatty acids of the two strains are C_16:0_, summed feature 3 (C_16:1_ω7c/C_16:1_ω6c), summed feature 8 (C_18:1_ω7c), and C_17:0_ cyclo. Transmission electron micrographs of strains 142053^T^ and 142359^T^ are shown in Supplementary Figsures S6, S7, respectively.

**Table 2:** Biochemical characteristics of strain 142053^T^, 142359^T^, and other species within the genus *Kluyvera.*

Characteristic	142053^T^	142359^T^	*K. sichuanensis*	*K. ascorbata*	*K. cryocrescens*	*K. intermedia*	*K. georgiana*
Motility	+	+	+	+	+	+	+
Indole production	+	+	−	+	+	−	+
Vojes–Projkauer reaction	−	−	−	−	−	+	−
Citrate utilization	+	+	+	+	+	+	+
H_2_S production	−	−	−	−	−	−	−
KCN growth in	+	+	+	+	+	+	+
Malonate utilization	+	+	+	+	+	+	−
NO_3_ → NO_2_	+	+	+	+	+	+	ND
ONPG test	+	+	−	+	+	+	+
Oxidase	−	−	−	−	−	−	ND
Catalase	+	+	−	+	+	+	ND
Lipase	W	W	−	−	−	−	−
Lysine decarboxylase	+	+	+	+	−	−	+
Arginine dihydrolase	+	+	−	−	−	−	−
Acid production from:							
Sucrose	+	+	−	+	+	+	+
Dulcitol	−	−	−	−	−	+	+
ᴅ-sorbitol	+	+	+	−	−	+	−
ᴅ-arabitol	−	+	−	−	−	−	−
Raffinose	+	+	−	+	+	+	+
Amygdalin	W	W	+	ND	ND	+	+
Glycerol	W	W	−	+	+	+	+
ᴅ-mannose	+	+	−	+	+	ND	ND
ᴅ-galactose	+	+	+	+	+	ND	ND
Methyl α-ᴅ-glucopyranoside	+	+	+	+	+	+	ND
ᴅ-lactose	W	+	+	+	+	+	+

Data for other *Kluyvera* species are from references (Farmer et al., 1981; Pavan et al., 2005; Liu et al., 2020). Only key results of biochemical reactions are shown in the table. The following tests gave identical results for the seven species: acid production from ʟ-rhamnose (+), ᴅ-maltose (+), ᴅ-xylose (+), ᴅ-trehalose (+), ᴅ-cellobiose (+), ᴅ-melibiose (+), ᴅ-glucose (+), mannitol (+), salicin (+), ʟ-arabinose (+), Aesculin (+), decarboxylase (+), erythritol (−), adonitol (−), inositol (−), Phenylalanine deaminase (−), Urease (−), Deoxyribonuclease (−), Gelatinase (−), Ornithine (−), ND, not done; +, positive; −, negative; W, weak positive.

The two strains were resistant to ampicillin but susceptible to ampicillin-sulbactam, aztreonam, ceftriaxone, ceftazidime, cefepime, cefotaxime, ciprofloxacin, imipenem, meropenem, piperacillin-tazobactam, amikacin, colistin, and tigecycline. Strain 142053^T^ was susceptible to cefuroxime but resistant to sulfamethoxazole-trimethoprim, while strain 142359^T^ showed the opposite pattern (Supplementary Table S2). Like other *Kluyvera* species, both strains possess an intrinsic chromosomal *bla*_CTX-M_ gene. Strain 142053^T^ encodes CTX-M-3, which is closely related to CTX-M-37 (differing by three amino acids) from *Kluyvera* genomospecies 5 (now to be considered *K. huaxiensis*) (Rodríguez et al., 2021), whereas strain 142359^T^ harbors the novel variant CTX-M-283 (GenBank accession no. PV575350). In addition, strain 142053^T^ also carries fosfomycin resistance gene *fosA3*, sulfonamide-resistance gene *sul1*, trimethoprim-resistance gene *dfrA21,* and a plasmid with an IncFIB (pHCM2) replicon, which likely accounts for the last two resistance markers.

### Description of *Kluyvera huaxiensis* sp. nov.

*Kluyvera huaxiensis* (hua. xi. en’sis N. L. fem. Adj. *huaxiensis*, referring to West China [“Huaxi” in Chinese], where the type strain was recovered).

The strain 142053^T^ is Gram-negative, facultatively anaerobic, motile, and non-spore-forming rods, measuring 0.6–0.8 μm in width and 1.0–2.5 μm in length. After 12-h incubation on nutrient agar, colonies are round, smooth, convex, and white. Growth occurs at 8–42 °C (optimum, 28–37 °C), pH 5.0–9.0 (optimum, 6.0–8.0), and NaCl concentrations from 0 to 5% (w/v) (optimum, 0–4%). Strain 142053^T^ is positive for indole production, nitrate reduction, citrate utilization, and enzymatic activities including lysine decarboxylase, arginine dihydrolase, ornithine decarboxylase, *β*-galactosidase (ONPG), and catalase. The strain assimilates aesculin, amygdalin, ᴅ-cellobiose, ᴅ-glucose, ᴅ-galactose, ᴅ-lactose, ᴅ-maltose, ᴅ-melibiose, ᴅ-sorbitol, ᴅ-trehalose, ᴅ-xylose, ʟ-arabinose, ʟ-rhamnose, mannitol, malonate, glycerol, methyl *α*-ᴅ-glucopyranoside, and salicin. It is negative for acetoin production (Voges-Proskauer test), DNase activity, H_2_S production, oxidase activity, phenylalanine deaminase activity, gelatinase, and urease. No utilization is observed for adonitol, dulcitol, erythritol, or inositol. Notably, the strain exhibits weak lipase activity and is positive for arginine dihydrolase, while ᴅ-arabitol metabolism is not detected. The major cellular fatty acids are C_16:0_ (26.5%), summed feature 3 (C_16:1_ω7c/C_16:1_ω6c, 24.4%), summed feature 8 (C_18:1_ω7c, 18.0%) and C_17:0_ cyclo (9.5%). The genomic DNA G + C content is 53.49 mol%.

The type strain is 142053^T^ (= GDMCC 1.4845^T^ = JCM 37413^T^), isolated from a clinical sputum sample at West China Hospital, Chengdu, China. The GenBank accession number for the 16S rRNA gene sequence is PV388010, and the draft genome sequence has been deposited under JBEFLV000000000 at DDBJ/EMBL/GenBank.

### Description of *Kluyvera chengduensis* sp. nov.

*Kluyvera chengduensis* (cheng. du. en’sis N. L. fem. Adj. *chengduensis*, referring to Chengdu, China, where the type strain was recovered).

Cells of strain 142359^T^ are Gram-negative, facultatively anaerobic, motile, non-spore-forming rods, measuring 0.6–0.8 μm in width and 1.0–1.5 μm in length. After 12-h incubation on nutrient agar, colonies are round, smooth, convex, and white. Growth occurs at 8–42 °C (optimum, 28–37 °C), pH 5.0–9.0 (optimum, 6.0–8.0), and NaCl concentrations 0–5% (w/v) (optimum, 0–4%). Strain 142359^T^ is positive for indole production, nitrate reduction, citrate utilization, and the activities of lysine decarboxylase, arginine dihydrolase, ornithine decarboxylase, *β*-galactosidase (ONPG), and catalase. The strain assimilates aesculin, amygdalin, ᴅ-cellobiose, ᴅ-glucose, ᴅ-galactose, ᴅ-lactose, ᴅ-maltose, ᴅ-melibiose, ᴅ-sorbitol, ᴅ-trehalose, ᴅ-xylose, ʟ-arabinose, ʟ-rhamnose, mannitol, malonate, glycerol, methyl *α*-ᴅ-glucopyranoside, and salicin. It is negative for acetoin production (Voges–Proskauer), DNase activity, H_2_S production, oxidase activity, phenylalanine deaminase, gelatinase, and urease. The strain does not utilize adonitol, dulcitol, erythritol, or inositol. Additionally, it displays weakly positive lipase activity and is positive for arginine dihydrolase. Strain 142359^T^ can further metabolize ᴅ-arabitol. The major cellular fatty acids are C_16:0_ (24.9%), summed feature 8 (C_18:1_ω7c, 22.7%), summed feature 3 (C_16:1_ω7c/C_16:1_ω6c, 20.0%), and C_17:0_ cyclo (9.7%). The genomic DNA G + C content is 54.48 mol%.

The type strain is 142359^T^ (= GDMCC 1.5094^T^ = JCM 37737^T^), isolated from a clinical sputum sample at West China Hospital, Chengdu, China. The GenBank accession number for the 16S rRNA gene sequence is PV388012, and the draft genome sequence has been deposited under JBIQOI000000000 at DDBJ/EMBL/GenBank.

## Discussion

Strains 142053^T^ and 142359^T^ were preliminarily assigned to the *Kluyvera* species by MALDI-TOF. A maximum-likelihood tree of 265 *Enterobacteriaceae* core genes placed strains 142053^T^ and 142359^T^ within the *Kluyvera* clade, clearly separated from related genera. Phylogenomic analysis based on 1,668 *Kluyvera* core genes further resolved strain 142053^T^ and strain 142359^T^ into distinct branches, supporting their classification as two different species. Although the genus *Kluyvera* currently comprises only five validly published species, inclusion of five previously proposed but unvalidated genomospecies (1, 2, 3, 4/6, and 5) was necessary to provide a comprehensive phylogenomic framework for the genus. These genomospecies have been consistently recognized in previous taxonomic and genomic studies as distinct *Kluyvera* lineages, and therefore they were retained in the phylogenetic analysis (Liu et al., 2020; Rodríguez et al., 2021; Rodriguez and Gutkind, 2024). The analysis revealed that *K.* genomospecies 5 and *K. huaxiensis* clustered together and exhibited high *is*DDH and ANI values (Table 1; Figure 2), confirming that they represent the same species. Therefore, the entity previously designated as *K.* genomospecies 5 should be reclassified as *K. huaxiensis*. Notably, the genomic sequence named *Kluyvera* sp. STS39-E (accession no. GCA 040932435) likely represents a novel genomospecies within the genus *Kluyvera*. As for clinical significance, strain 142053^T^ was isolated from a patient with bilateral interstitial pulmonary fibrosis who did not exhibit any clinical manifestation of pneumonia, and hence it was considered as a colonizer. In contrast, strain 142359^T^ was recovered from a patient with pneumonia, which improved following treatment with piperacillin-tazobactam. However, as the same sputum sample also yielded *Klebsiella pneumoniae*, the clinical significance of strain 142359^T^ remains uncertain, and it may represent either a pathogen in a mixed infection or a colonizer in a patient with *K. pneumoniae*-associated pneumonia.

There are limitations of this study. First, only a single isolate was obtained for each of the two proposed species. Although both species are represented by at least two independent genomes, the phenotypic characterization based on a single isolate may not fully capture intraspecies variability. Second, phenotypic features of other *Kluyvera* species were derived from published studies, which may vary due to difference in experimental conditions. Third, polar lipids, quinones and whole-cell sugar compositions were not analyzed. As such, phenotypic traits used to distinguish the two proposed species from other *Kluyvera* species should be interpreted with caution.

Nevertheless, the core-genome phylogenetic analysis, together with chemotaxonomic data, clearly places strain 142053^T^ and 142359^T^ within the genus *Kluyvera*. The ANI and *is*DDH analyses conclusively demonstrate that these strains represent two novel species, for which the names *Kluyvera huaxiensi*s sp. nov. and *Kluyvera chengduensis* sp. nov. are proposed.

## Data Availability

The 16S rRNA gene sequences for strains 142053^T^ are deposited in GenBank under accession no. PV388010. Additionally, strain 142359^T^ has 16S rRNA gene accession no. PV388012. Genome assemblies for these strains are recorded under the accession nos. JBEFLV000000000 and JBIQOI000000000, respectively.

## Footnotes

1
https://tygs.dsmz.de/user_requests/new

2
https://github.com/tseemann/abricate

3
https://genomicepidemiology.org

4
http://www.eucast.org/
