# Supplementary material for: Reporting two novel Kluyvera species, Kluyvera huaxiensis and Kluyvera chengduensis, isolated from human sputa
Source: Front Microbiol. 2026 Jan 5;16:1683098. doi: 10.3389/fmicb.2025.1683098 (PMC12812546; doi:10.3389/fmicb.2025.1683098)
Supplement: Supplementary file 1 [file Table_1.DOCX]

| **Fatty acid** | 142053T | 142056T | GDMCC 1.1872T |
| --- | --- | --- | --- |
| C12:0 | 2.5 | 3.3 | 4.2 |
| C14:0 | 7.5 | 7.4 | 8.7 |
| C16:0 | 26.5 | 24.9 | 31.3 |
| C17:0 cyclo | 9.5 | 9.7 | 17.6 |
| Sum In Feature 2（C14:0 3-OH/C16:1 iso I） | 8.7 | 8.4 | 7.5 |
| Sum In Feature 3(C16:1ω7c/C16:1ω6c) | 24.4 | 20.0 | 11.3 |
| Sum In Feature 8 (C18:1ω7c) | 9.5 | 22.7 | 15.7 |

Table S1. Cellular fatty acid profiles of two novel strains: analysis and comparison with reference strain *K. sichuanensis* GDMCC 1.1872^T^

Table S2. Antimicrobial susceptibility results of strains 142053^T^ and 142359^T^

| **Antimicrobial** | **MIC** | | **R/S** | |
| --- | --- | --- | --- | --- |
|  | **142053** | **142359** | **142053** | **142359** |
| Meropenem | ＜0.0625 | ＜0.0625 | S | S |
| Imipenem | 0.125 | 0.125 | S | S |
| Ampicillin | 32 | 32 | R | R |
| Ceftriaxone | 0.064 | 1 | S | S |
| Ciprofloxacin | ＜0.0625 | ＜0.0625 | S | S |
| Ceftazidime | ＜0.0625 | 0.125 | S | S |
| Chloramphenicol | 2 | 2 | S | S |
| Cefepime | ＜0.0625 | 0.125 | S | S |
| Cefotaxime | 0.125 | 0.5 | S | S |
| Ampicillin-sulbactam | 0.5 | 2 | S | S |
| Cefuroxime | 4 | 32 | S | R |
| amikacin | 0.5 | 0.5 | S | S |
| aztreonam | ＜0.0625 | ＜0.0625 | S | S |
| colistin | 0.5 | 0.125 | S | S |
| piperacillin-tazobactam | 0.125 | 1 | S | S |
| tigecycline | 0.125 | 0.25 | S | S |
| sulfamethoxazole-trimethoprim | 304/16 | 4.75/0.25 | S | S |


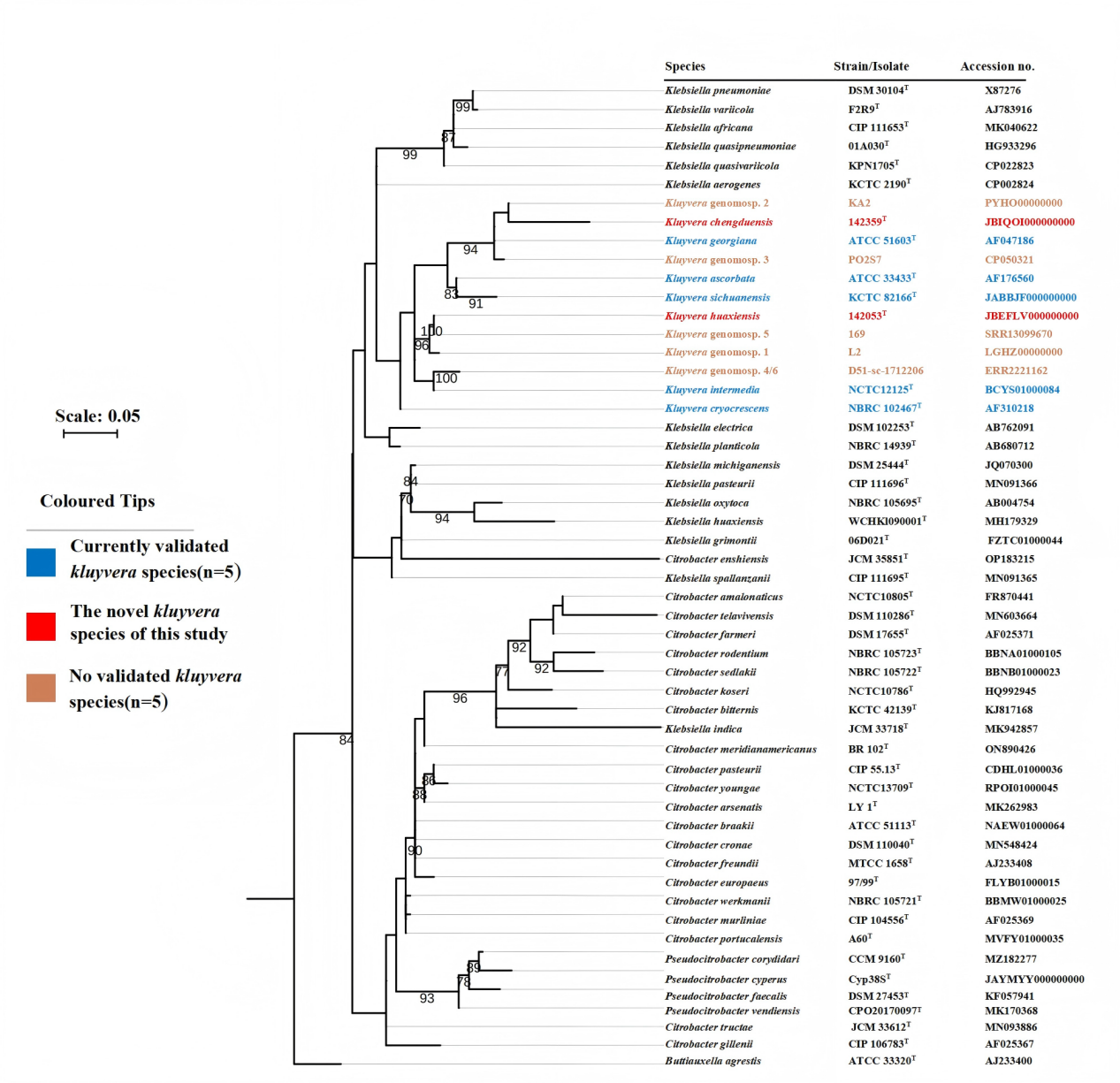


Fig. S1. Phylogenetic relationships of strain 142053^T^ and 142359^T^, other *Kluyvera* species, and closely related genera, based on 16S rRNA gene sequences. The tree was constructed using the maximum-likelihood method. Bootstrap values greater than 70% (based on 1,000 resamplings) are shown. Bar, 0.05 substitutions per nucleotide position.


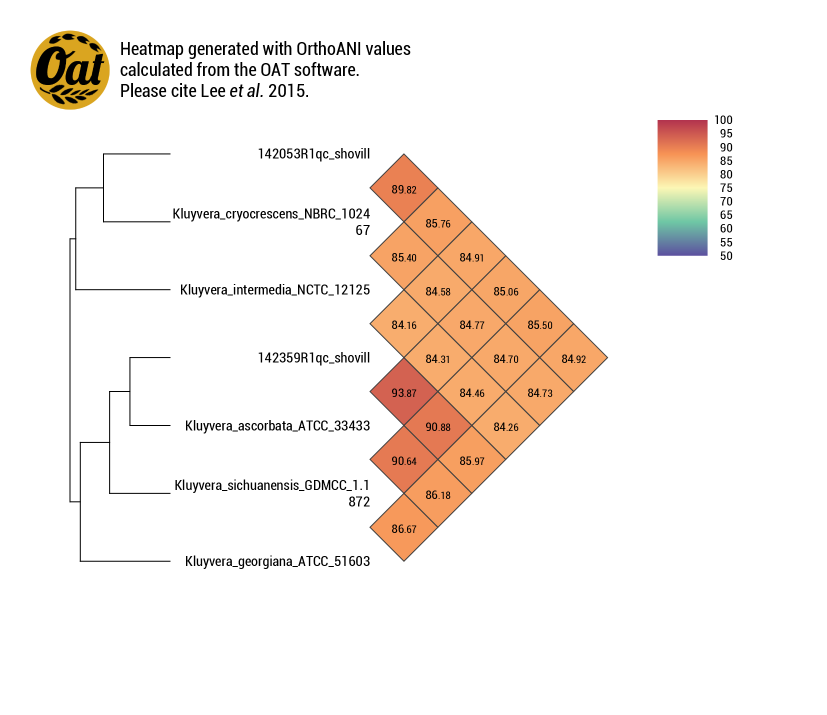


Fig. S2. The heatmap was generated based on OrthoANI to visualize the genomic relationships among strain 142053^T^, strain 142359^T^, and type strains of *Kluyvera* species.


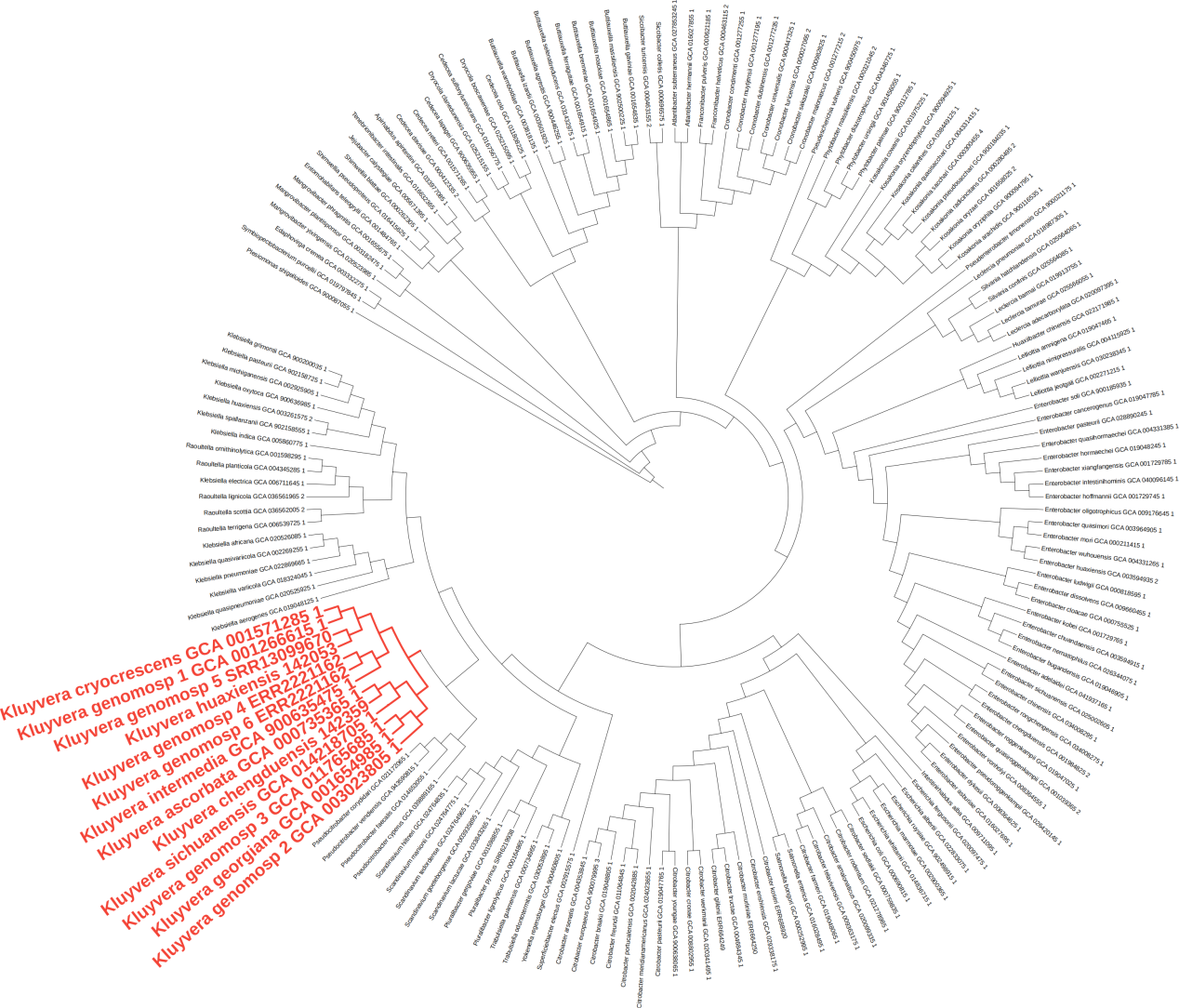


Fig. S3. Phylogenomic tree of strain 142053^T^, 142359^T^, and other genera in the family *Enterobacteriaceae*. The core genome tree was constructed via the neighbor-joining method based on 1,000 resamplings.


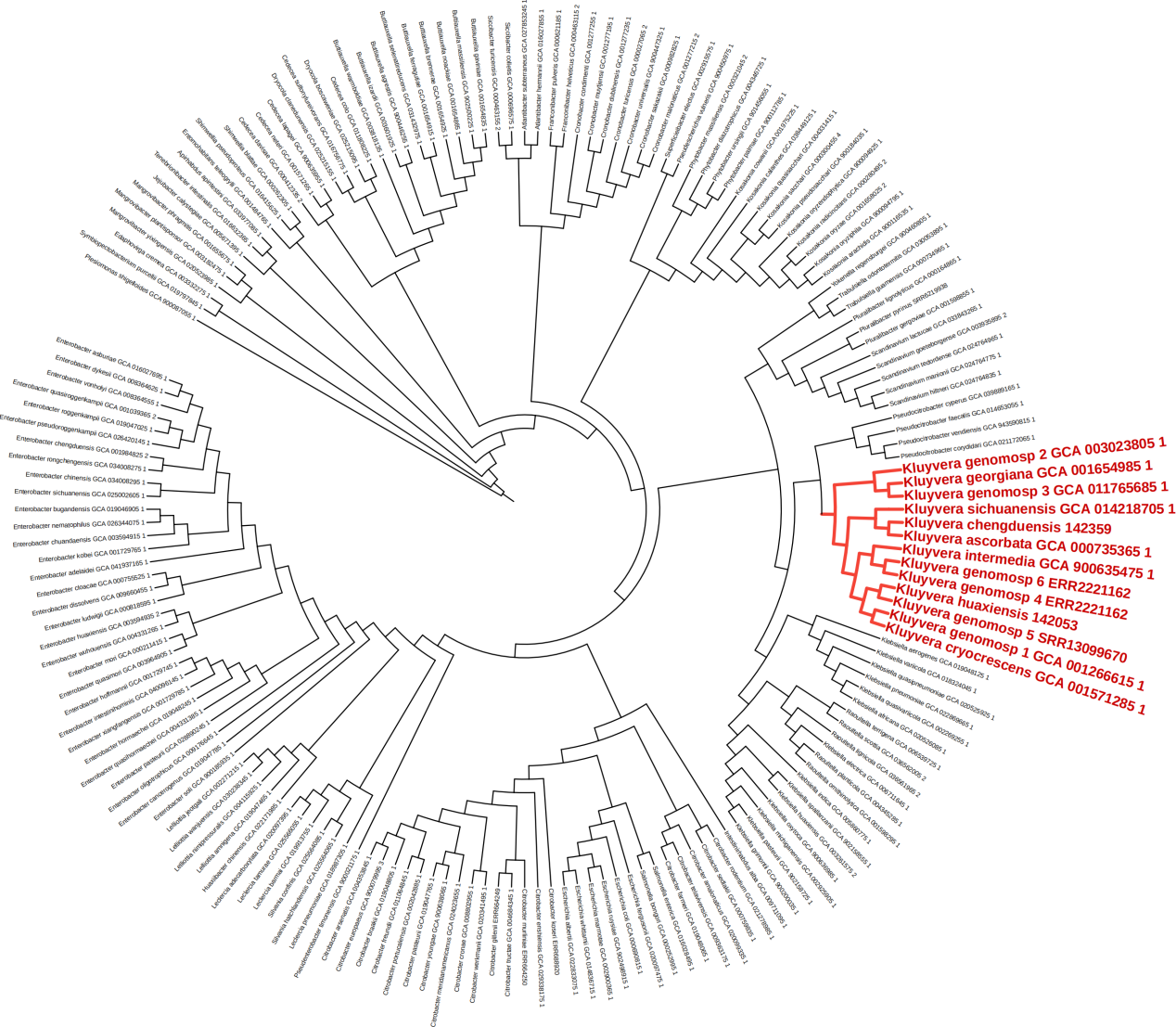


Fig. S4. Phylogenomic tree of strain 142053^T^, 142359^T^, and other genera in the family *Enterobacteriaceae*. The core genome tree was constructed via the maximum parsimony method based on 1,000 resamplings.
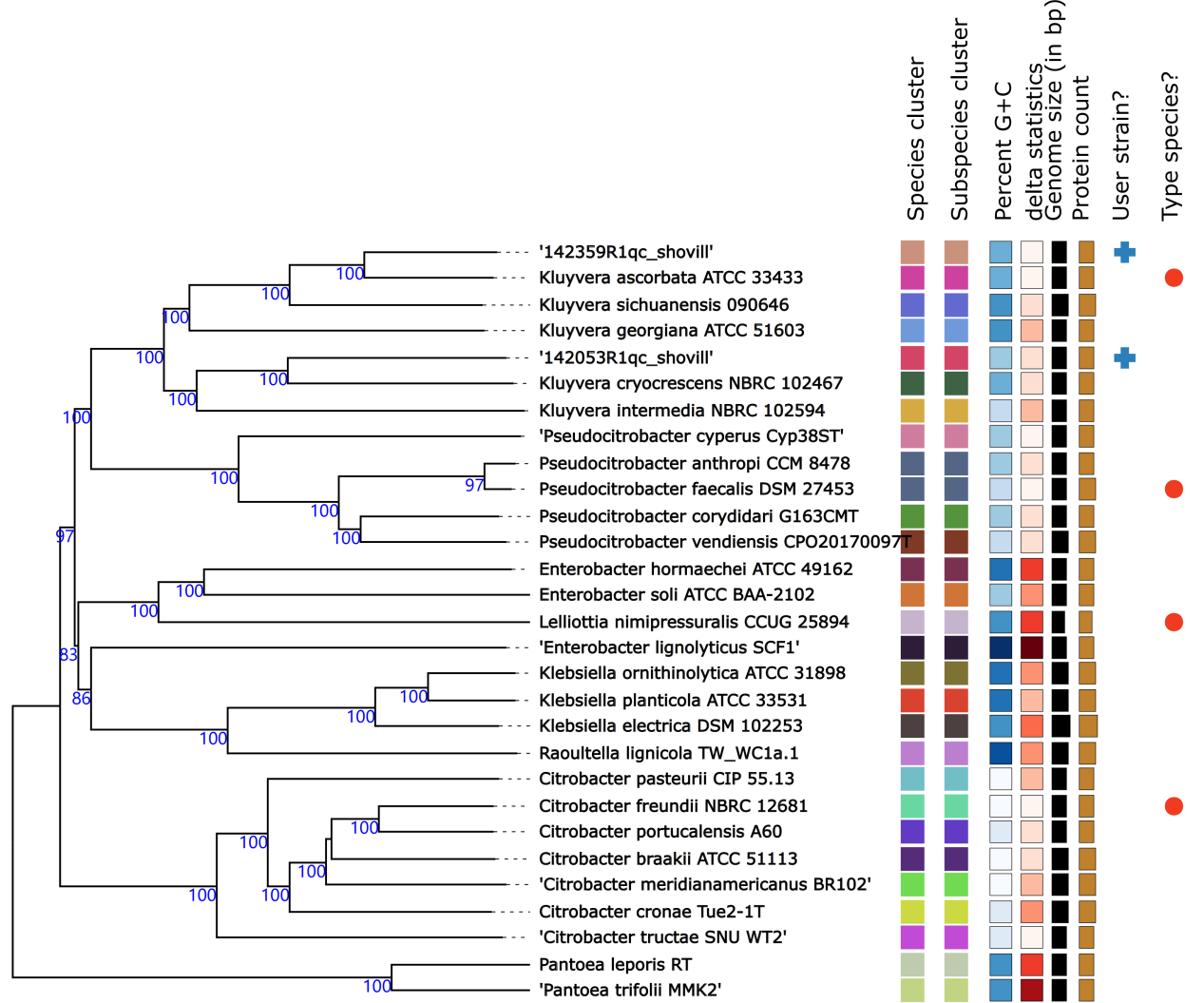


Fig. S5. The TYGS phylogenomic tree of strain 142053^T^ and 142359^T^


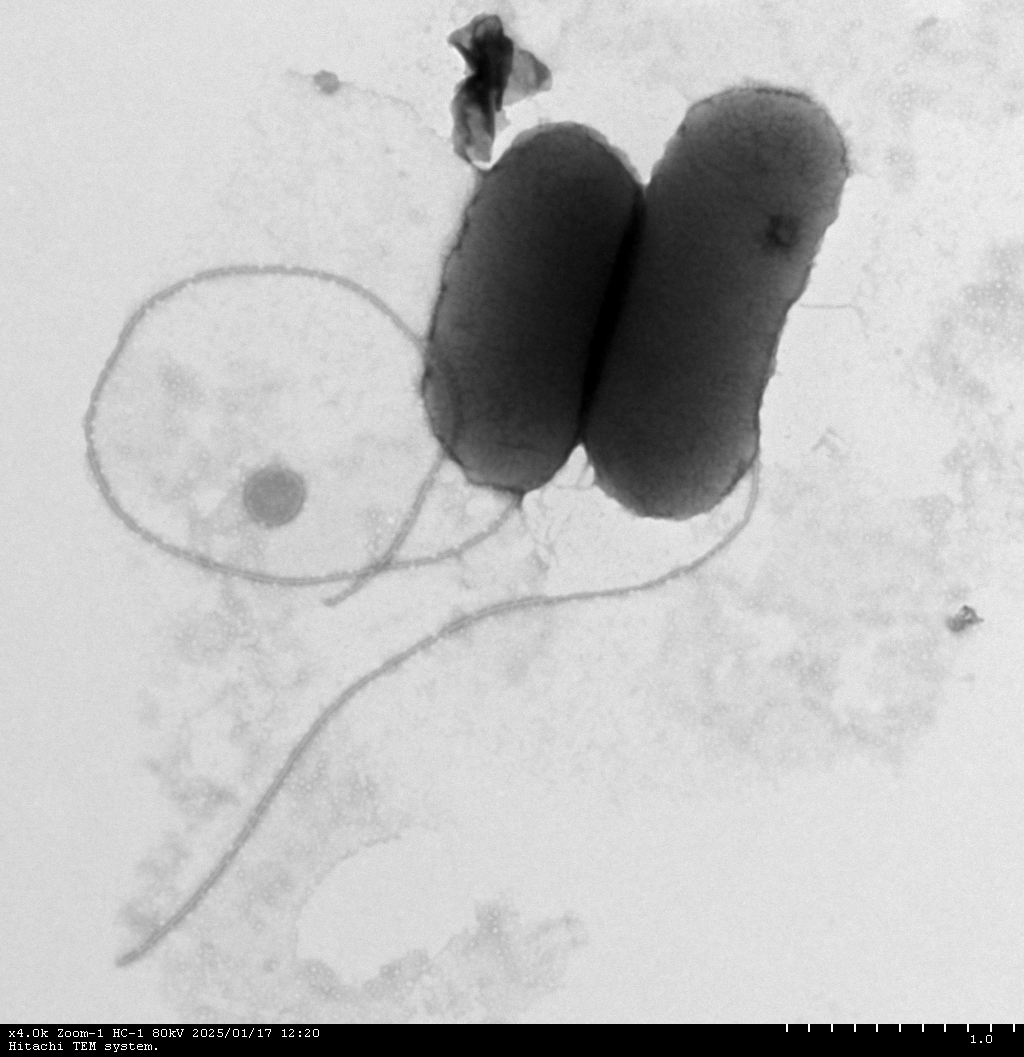


Fig. S6. Transmission electron micrograph of strain 142053^T^


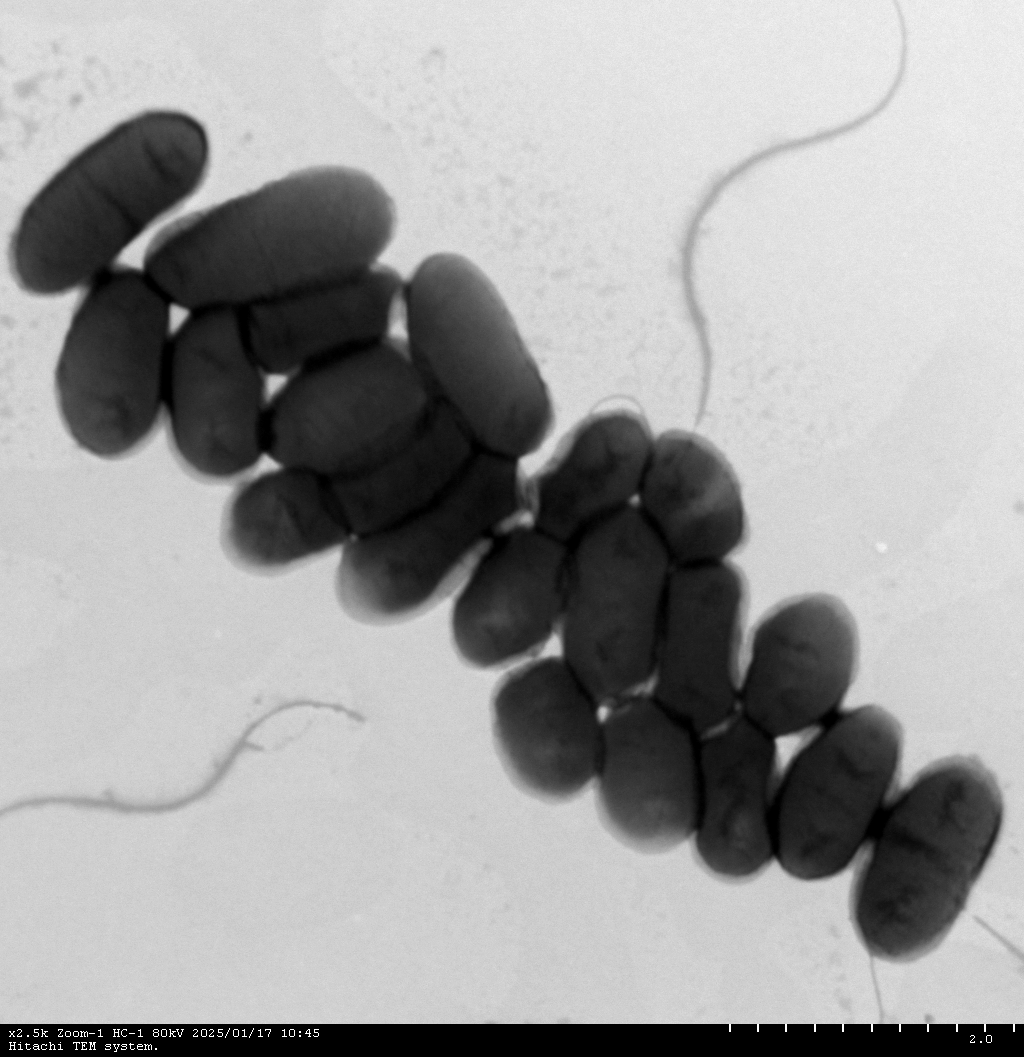


Fig. S7. Transmission electron micrograph of strain 142359^T^
